# Bioaugmentation of Atrazine-Contaminated Soil With *Paenarthrobacter* sp. Strain AT-5 and Its Effect on the Soil Microbiome

**DOI:** 10.3389/fmicb.2021.771463

**Published:** 2021-12-08

**Authors:** Weibin Jia, Ning Li, Tunan Yang, Weixian Dai, Jiandong Jiang, Kai Chen, Xihui Xu

**Affiliations:** Department of Microbiology, Key Laboratory of Environmental Microbiology for Agriculture, Ministry of Agriculture, College of Life Sciences, Nanjing Agricultural University, Nanjing, China

**Keywords:** atrazine, bioaugmentation, phytotoxicity, *Paenarthrobacter* sp. AT-5, soil microbiome

## Abstract

Atrazine, a triazine herbicide, is widely used around the world. The residue of atrazine due to its application in the fore-rotating crop maize has caused phytotoxicity to the following crop sweet potato in China. Bioaugmentation of atrazine-contaminated soil with atrazine-degrading strains is considered as the most potential method to remove atrazine from soil. Nevertheless, the feasibility of bioaugmentation and its effect on soil microbiome still need investigation. In this study, *Paenarthrobacter* sp. AT-5, an atrazine-degrading strain, was inoculated into agricultural soils contaminated with atrazine to investigate the bioaugmentation process and the reassembly of the soil microbiome. It was found that 95.9% of 5 mg kg^−1^ atrazine was removed from the soils when inoculated with strain AT-5 with 7 days, and the phytotoxicity of sweet potato caused by atrazine was significantly alleviated. qRT-PCR analysis revealed that the inoculated strain AT-5 survived well in the soils and maintained a relatively high abundance. The inoculation of strain AT-5 significantly affected the community structure of the soil microbiome, and the abundances of bacteria associated with atrazine degradation were improved.

## Introduction

Atrazine is one of the photosystem-II (PSII)-inhibiting herbicides, which destroy the chloroplast light systems of plants, causing the plants to become chlorotic and finally wither and die. Atrazine is widely used in the prevention and control of broad-leaved weeds in maize, pineapple, sorghum, and sugar cane (Strong et al., 2000). Since atrazine was first put on the market in the 1950s, it has become the second largest applied pesticide in the world because of its high efficiency and low cost (Singh et al., 2018; Cao et al., 2021). Atrazine has a long half-life in soils, varying from approximately 60 days to over 1 year (Smith et al., 2005). With the wide application of atrazine, the residue of atrazine in the soil has caused great concern. Atrazine has been frequently detected in environments with concentrations as high as 250 mg kg^−1^ in soil (Chiaia-Hernandez et al., 2017), 30 μgL^−1^ in groundwater (Cerejeira et al., 2003), and 5 μgL^−1^ in surface water (Ge et al., 2010). As a potent endocrine disruptor, atrazine shows potential risk for endocrine health and immune disruption (Brodkin et al., 2007), nervous system damage (Rusiecki et al., 2004), and reproductive cancers in laboratory rodents and humans (Fan et al., 2007). Furthermore, the residual atrazine in soils also causes phytotoxicity to subsequent crops, such as soybean (Soltani et al., 2011; Zhang et al., 2021), sorghum, oat, wheat, and sweet potato (Lima et al., 2020). Therefore, effective removal of atrazine residues from soil is of great importance.

Atrazine in environments can be dissipated by the following methods: oxidative degradation by zero-valent metals (Diao et al., 2020), photolysis (Souza et al., 2014), advanced oxidation processes (Morales-Perez et al., 2016), or bioremediation. As an eco-friendly, efficient, and low-cost method, bioremediation has been proposed as the most promising method to remove atrazine from contaminated-sites. Microorganisms capable of degrading atrazine have been reported since 1995, such as *Pseudomonas* sp. strain ADP, which is the first isolated atrazine-mineralizing strain (de Souza et al., 1998). Subsequently, more and more atrazine-degrading strains have been isolated, including *Nocardioides* sp. SP12 (Piutti et al., 2003), *Arthrobacter* sp. GZK-1 (Getenga et al., 2009), *Arthrobacter* sp. AK-YN10 (Sagarkar et al., 2016) and so on (Huang et al., 2017). To date, two types of atrazine-degrading bacteria have been described: (i) capable of completely mineralizing atrazine, harboring the genes of *atzA*/*trzN*, *atzB*, *atzC*, *atzD*, *atzE*, and *atzF* (Piutti et al., 2003), and (ii) capable of transforming atrazine into cyanuric acid, harboring the genes of *trzN*, *atzB* and *atzC* (Hernandez et al., 2011). Generally, gram-positive bacteria initiate the atrazine degradation *via* the hydrolysis reaction catalyzed by TrzN, while gram-negative bacteria catalyze the reaction by AtzA (Omotayo et al., 2011; Huang et al., 2017).

In previous studies, bioaugmentation has been proposed for atrazine degradation by inoculating atrazine-degrading bacteria to soils (Fadullon et al., 1998). Successful bioaugmentation requires the survival of the inoculated strains and keeping metabolic activity (Gomes et al., 2005). To date, the quantitative analysis of specific gene based on real-time PCR has been used for evaluating the genetic stability and survival of inoculated strains in soil samples (Chi et al., 2013). However, the interactions between the contaminants, inoculated strains, and the soil indigenous microbial communities remain largely unknown. In addition, the soil microbiome largely affects the productivity and stability of agroecosystems (van der Heijen, 2008). High-throughput sequencing has been successful in exploring the complex dynamic changes of microbial communities (Liu et al., 2020). Therefore, revealing the microbial community in soils responding to atrazine and its degrading strains will help to understand the underlying mechanism of bioaugmentation and improve the bioremediation efficiency.

In this study, an atrazine-degrading strain, *Paenarthrobacter* sp. AT-5 (formerly *Arthrobacter* sp. strain AT-5; Busse, 2016) was inoculated to evaluate the bioaugmentation processes in agricultural soils contaminated with atrazine (Xu et al., 2019). Real-time quantitative PCR (qRT-PCR) was used for calculating initial functional gene (*trzN*) to evaluate the stability and fate of strain AT-5 during the bioaugmentation process. At the same time, the alleviation of phytotoxicity of sweet potato by strain AT-5 was also investigated. More importantly, our study also aims to explore the dynamic changing process of soil microbiome during bioaugmentation. This study is helpful to understand the change of community structure of microbiome during bioaugmentation and provides a theoretical guidance for development of enhanced bioremediation strategies for atrazine-contaminated soils.

## Materials and Methods

### Soil Characteristics and Preparation of Inoculum

Soil samples were collected from a maize field (0–10 cm depth) in Jiangsu Province, China, after maize was harvested. Mixed and homogenized soil samples were passed through a 2-mm sieve and then stored at 4°C before further use. The chemical and physical properties of the soil were determined by standard methods (Nelson and Sommers, 1996) and summarized in Supplementary Table S1.

*Paenarthrobacter* sp. strain AT-5, an atrazine-degrading strain isolated previously in our lab (Liu et al., 2019; Xu et al., 2019), was used for bioaugmentation. Strain AT-5 was cultured in Luria-Bertani (LB) medium at 30°C and pH 7.0 until the exponential phase. Then, the cells were harvested through centrifugation (6,000*g* for 6 min) and washed with sterilized minimal salt medium for three times.

### Phytotoxicity of Sweet Potato Seedlings Caused by Atrazine Residues in Soil

To study the phytotoxicity of subsequent crop caused by atrazine residues in soil, the “Su 22” sweet potato seedlings (obtained from Jiangsu Academy of Agricultural Sciences, China) were selected. Soils with atrazine residues were prepared by supplementing atrazine (dissolved in methanol, Sigma-Aldrich, Shanghai, China; 98% purity) to soils at five different final concentrations (10 mg kg^−1^, 5 mg kg^−1^, 1 mg kg^−1^, 0.5 mg kg^−1^, and 0.2 mg kg^−1^, dry soil weight). The atrazine-spiked soils (250 g, dry weight) were transferred to three pots, respectively. The “Su 22” sweet potato seedlings were planted into pots and put in a growth chamber (Jiangnan, Ningbo, China) at 28/25°C with an 18-h light/6-h dark cycle. During the incubation, water was added every other day to keep the soil moisture content at 40%. The growth status of sweet potato seedlings was observed and recorded at 0, 1, 3, 7, 14, and 21 days, and three replicates were set for each treatment.

### Bioaugmentation of Atrazine-Contaminated Soil With Strain AT-5

Atrazine-spiked soils were prepared as described above at a final atrazine concentration of 5 mg kg^−1^ (dry soil weight), and the carrier solvent methanol was removed through evaporation. The microcosm treatments were set as follows: (i) Control: equivalent amount of methanol and ddH_2_O were added into native soil, (ii) Atr: native soil spiked with atrazine, (iii) Atr-Bio: atrazine-spiked soil with inoculation of strain AT-5 (1.0 × 10^7^CFU/g dry soil), (iv) Bio: native soil with inoculation of strain AT-5 (1.0 × 10^7^CFU/g dry soil), and (v) Sterilized soil: atrazine-spiked soil autoclaved three times (121°C, 20 min). The treated soils were sorted into plastic pots (700 g dry soil per pot) and then incubated at 28/25°C with an 18-h light/6-h dark cycle in a growth chamber for 14 days. The soil moisture content was kept at 30% of the soil water holding capacity (WHC), and the lost water was replenished by weighing samples every day. Soils (20 g per pot) were non-destructively sampled at 0, 1, 3, 5, 7, and 14 days for atrazine detection and bacterial community analysis. Three replicates were set for each treatment.

The remaining soils of the atrazine-spiked soil (Atr) and atrazine-spiked soil with inoculation of strain AT-5 (Atr-Bio) at 14 d were planted with sweet potato seedlings to verify whether the phytotoxicity caused by atrazine was alleviated by bioaugmentation with strain AT-5. The growth status of sweet potato seedlings was measured as described in section “Phytotoxicity of Sweet Potato Caused by Atrazine and Its Alleviation by Bioaugmentation”.

### Extraction and Analysis of Atrazine in Soil

At each sampling time, 20 g soil (dry weight) was collected and extracted three times with 60 ml dichloromethane by horizontally mixing for 2 h at 30°C and ultra-sonication for 30 min. The extracts were concentrated by rotary evaporation, dried with nitrogen, and then resolved in 1 ml of methanol. Using this method, the extraction efficiency of atrazine in soil was 79.9%. The impurities in the extracts were removed by filtering through a 0.22-μm membrane. Atrazine was detected through high-performance liquid chromatography (HPLC; UltiMate 3,000 RSLC; Thermo Fisher Scientific, United States) using a reversed-phase C_18_ separation column (250 mm × 4.6 mm × 5 μm; Thermo Fisher Scientific, Waltham, MA, United States). The mobile phase consisted of 20% water and 80% methanol (vol/vol), and the flow rate was 0.8 ml min^−1^. The column temperature was 30°C, and the injection volume was 20 μl. Atrazine was detected at 220 nm. Under these conditions, the retention time (Rt) for atrazine was 5.97 min.

### DNA Extraction, Sequencing, and Quantitative Real-Time PCR

Soil total DNA was extracted from the soil sample (0.5 g, dry weight) using a Fast DNA SPIN Kit for Soil (MP Biomedicals, United States). The V4 region of the 16S rRNA gene was amplified using the primer set 515F and 806R as described previously (Jia et al., 2021a). The amplified 16S rRNA genes were sequenced by Biozeron Biological Technology Co. Ltd. (Shanghai, China) on an Illumina HiSeq 2,500 platform.

The numbers of the inoculated strain AT-5 in soil were estimated by quantifying the copy numbers of *trzN* gene (encoding the initial hydrolase for atrazine degradation) through quantitative real-time PCR (qRT-PCR) with primers (RT-trzNF: GCAGCGTTTCACGGACAA, RT-trzNR: AGGAGCGACTGGAGGAGGAC; 235bp; Sajjaphan et al., 2004). The *trzN* fragment was amplified from the DNA of strain AT-5 with primers (trzN-F: ATGATCCTGATCCGCGGACT, trzN-R: CTACAAGTTCTTGGGAATGA; 1383bp) and cloned into pMD™19-T Vectors (TaKaRa, Dalian, China). The recombinant plasmid was used as the standard for quantitative analysis. The concentrations of the recombinant plasmid were detected on a NanoDrop ND-2000 spectrophotometer (ND2000, Thermo Scientific, DE, United States), and then the copy numbers of inserted *trzN* gene were calculated. Tenfold serial dilutions of the concentration-known recombinant plasmid (in triplicate) were used in the qRT-PCR assay to generate an external standard curve. The qRT-PCR was performed on the Applied Biosystems 7500 Fast real-time PCR system (Applied Biosystems, USA) with SYBR Premix Ex Taq II (Tli RNase H Plus; TaKaRa).

### Sequence Analysis of 16S rRNA Gene

After sequencing, the raw reads were merged using FLASH (Magoc and Salzberg, 2011). The merged reads were quality-filtered using QIIME, and then, effective tags were clustered into operational taxonomic units (OTUs) with a 97% similarity cutoff (Caporaso et al., 2010; Edgar, 2013). The species richness and diversity, including Chao1, Shannon, and Simpson index (1-lambda), were estimated through α-diversity analysis, using QIIME (Caporaso et al., 2010). β-diversity analysis was used to evaluate the similarity of bacterial communities in different treatments (Caporaso et al., 2010). The weighted UniFrac distances were calculated using the QIIME pipeline, and principal coordinates analysis (PCoA) was performed with R using the library “vegan.” Potential biomarkers were identified through linear discriminant analysis (LDA) with effect size (LEfSe; Segata et al., 2011). The Molecular Ecological Network Analysis (MENA) pipeline was used to construct the co-occurrence patterns of bacterial communities (Das and Mukherjee, 2007).

### Data Availability

The sequencing data involved in this manuscript are available at NCBI under BioProject ID PRJNA765205.

## Results

### Removal of Atrazine by Inoculation of Strain AT-5 and Its Dynamic Abundance in the Soil

**Figure 1:**
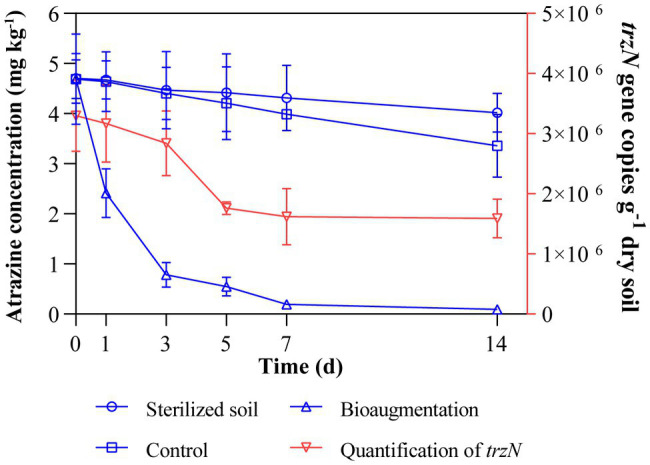
Dissipation of atrazine in various soil samples with time and qRT-PCR quantification of *trzN* in inoculated soil microcosms. Control: native soil spiked with atrazine; Sterilized soil: sterilized soil spiked with atrazine; Bioaugmentation: atrazine-spiked soil with inoculation of strain AT-5; Quantification of *trzN*: *trzN* gene copies (g^−1^ dry soil).

In the sterilized soil, only 14.5% of atrazine disappeared at the end of incubation (14 days), showing that abiotic factors have limited contributions to atrazine dissipation (Figure 1). Compared to that in the sterilized soil, the concentration of atrazine in bioaugmentation treatment (Atr-Bio) was significantly reduced. The removal rates of atrazine in the atrazine treatment (Atr) and bioaugmentation treatment (Atr-Bio) were 28.3 and 97.9% at 14 days, respectively. In addition, the DT_50_ and DT_90_ values of atrazine in Atr-Bio treatment were 1.2 and 3.9 days, respectively. These results showed that indigenous microorganisms contributed a little to atrazine removal and strain AT-5 dominated the atrazine removal in the soil (*p* < 0.01).

The abundance of the inoculated strain AT-5 in the soil was calculated by quantifying the copy numbers of the initial hydrolase gene *trzN* by qRT-PCR (Figure 1). The amplification efficiency of PCR threshold standard curve was 98.4%. In the bioaugmentation treatment, the copy numbers of *trzN* gene decreased within the first 7 days and then kept relatively stable (the same order of magnitude at 10^6^ copies g^−1^ dry soil) till 14 days, while the *trzN* gene could not be amplified with the DNA template extracted from the original soil without any treatments. These results indicated that the inoculated strain AT-5 could survive well in the soil for a period of time (14 days).

### Phytotoxicity of Sweet Potato Caused by Atrazine and Its Alleviation by Bioaugmentation

**Figure 2:**
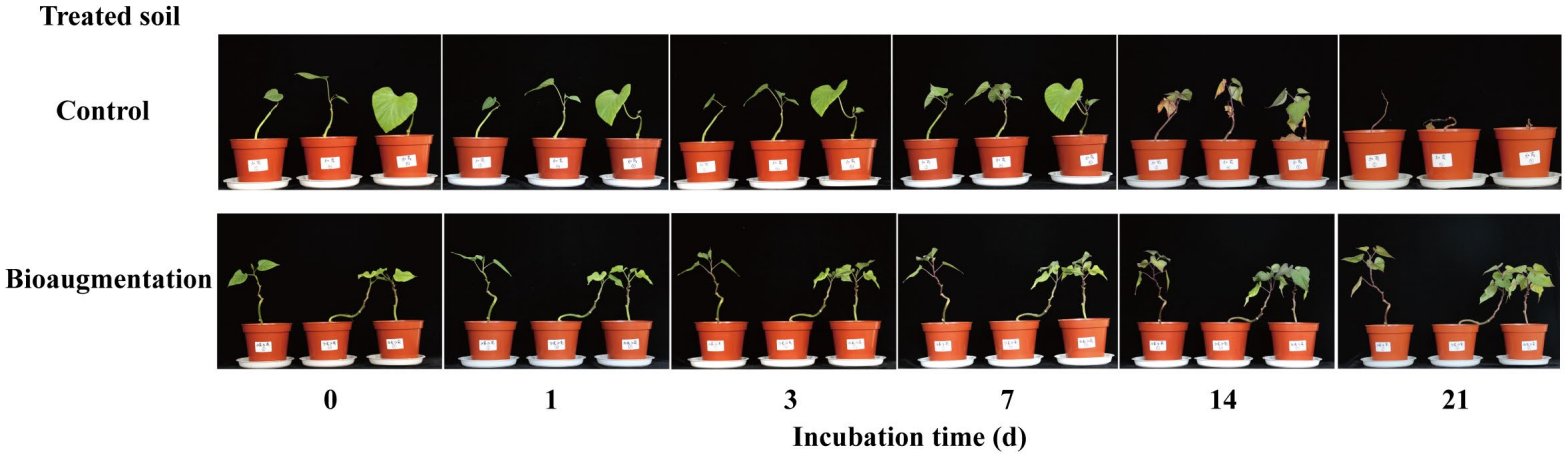
The phytotoxicity of atrazine on sweet potato seedlings alleviated by bioaugmentation. Control: native soil spiked with atrazine after incubated 14 days, and the concentration of atrazine remaining in the control soil was 3.36 ± 0.62 mg kg^−1^. Bioaugmentation: atrazine-spiked soil with inoculation of strain AT-5 after incubated 14 days, and the concentration of atrazine remaining in the bioaugmentation soil was 0.09 ± 0.002 mg kg^−1^. Three replicates were set for each treatment.

Both high and low concentrations of atrazine in the soil caused phytotoxicity to sweet potato seedlings (Supplementary Figure S2). With a low concentration of atrazine (0.2 mg kg^−1^) in the soil, the sweet potato seedlings survived within the 21-day cultivation period, but phytotoxicity phenomena, such as leaf yellowing, were still observed. These results suggested that sweet potato seedlings were sensitive to atrazine, and even low concentrations of atrazine in the soil would affect the planting of subsequent crops. Therefore, the remaining soils of the atrazine-spiked soil (Atr) and atrazine-spiked soil with inoculation of strain AT-5 (Atr-Bio) at 14 d were again planted with sweet potato seedlings. The concentrations of atrazine remaining in Atr and Atr-Bio soils were 3.36 ± 0.62 and 0.09 ± 0.002 mg kg^−1^, respectively. The atrazine-spiked soil (Atr) caused serious phytotoxicity to sweet potato seedlings, and sweet potato seedlings died at 21 days. However, the sweet potato seedlings grew well in the atrazine-spiked soil with inoculation of strain AT-5 (Atr-Bio; Figure 2). These results further showed that bioaugmentation with strain AT-5 was effective in the removal of atrazine from soil and could prevent the damage of atrazine residues to subsequent crops.

### Effects of Bioaugmentation and Atrazine Application on Soil Microbiome

**Figure 3:**
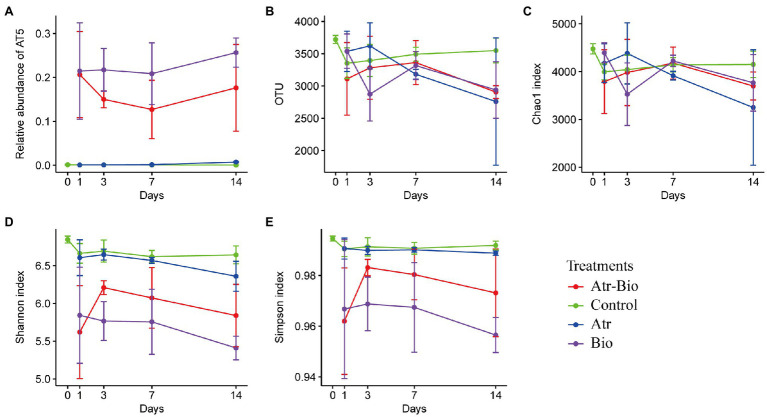
Richness estimators and diversity indices of bacterial communities. **(A)** relative abundance of strain AT-5; **(B)** observed OTU numbers; **(C)** Chao1 indices; **(D)** Shannon indices; **(E)** Simpson indices (1-lambda). Control: equivalent amount of methanol and ddH_2_O were added into native soil; Atr: native soil spiked with atrazine; Atr-Bio: atrazine-spiked soil with inoculation of strain AT-5; Bio: native soil with inoculation of strain AT-5.

The changes of the soil bacterial community at 0, 1, 3, 7, and 14 days in different treatment were investigated by sequencing of the 16S rRNA-amplicons. For α-diversities, the control treatment (methanol and ddH_2_O application) presented higher community richness (represented by higher Chao1 indices and observed OTU numbers) and diversity (indicated by Shannon and Simpson indices) than the Atr-Bio treatment (atrazine-spiked soil with inoculation of strain AT-5) and the Bio treatment (native soil with inoculation of strain AT-5; Figure 3). In addition, the application of atrazine without inoculation (Atr treatment) reduced the community richness, while had little effect on community diversity (Figure 3). These results indicated that the inoculation of degrading strain AT-5 significantly affected the bacterial community richness and diversity in soil.

**Figure 4:**
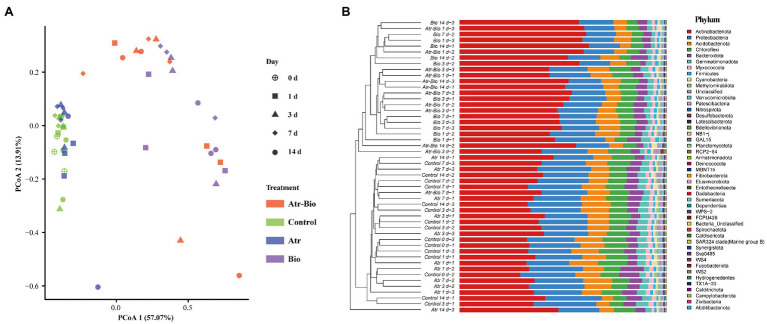
Effects of the different treatments on the bacterial community structure. **(A)** principal coordinates analysis (PCoA) with Bray–Curtis distances of bacterial communities; **(B)** Hierarchical clustering based on relative abundances of bacterial phylum in the soil samples. Control: equivalent amount of methanol and ddH_2_O were added into native soil; Atr: native soil spiked with atrazine; Atr-Bio: atrazine-spiked soil with inoculation of strain AT-5; Bio: native soil with inoculation of strain AT-5.

Principal coordinates analysis (PCoA) and hierarchical cluster analysis were used to investigate the influences of inoculation of strain AT-5 and atrazine application on soil bacterial communities. The soil samples were grouped into two distinct clusters based on different treatments and time: inoculation treatments (Atr-Bio and Bio) and non-inoculation treatments (Control and Atr; Figure 4A). In addition, the hierarchical cluster analysis also reflected the sample separation, presenting a greater distance between the inoculation treatments and other treatments (Supplementary Figure S5). These results showed that inoculation of strain AT-5 dominated the changes of bacterial community diversity and structure in the soil, while atrazine application nearly had no effect on bacterial community.

### Bacterial Abundance and Composition

In all treatments, the bacterial phyla with higher abundances were Actinobacteriota, Proteobacteria, Acidobacteriota, Chloroflexi, and Bacteroidota (Figure 4B) and specifically the bacterial genera were *Paenarthrobacter*, *Pseudarthrobacter* and *Nocardioides* (Supplementary Figure S6). The genus *Paenarthrobacter* was the dominant genus in inoculation treatments, and the relative abundances of *Paenarthrobacter* in the Atr-Bio treatment kept relatively stable (21.1, 15.3, 13.0, and 17.9% at 1, 3, 7, and 14 days, respectively). These results combined with the qRT-PCR data showed that the inoculated *Paenarthrobacter* sp. strain AT-5 stably survived in the soil (Figures 1, 3A; Supplementary Figure S6).

**Figure 5:**
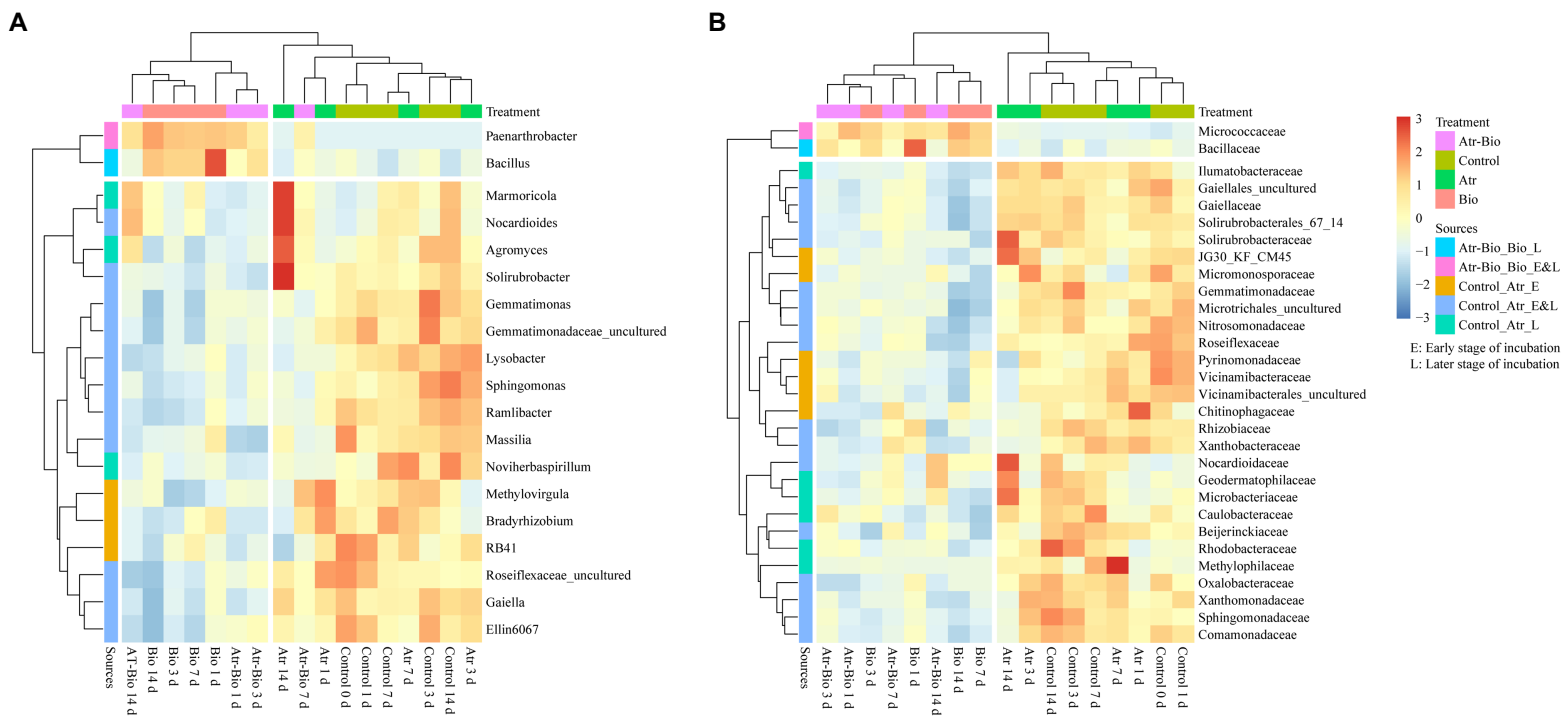
Heatmaps showing relative abundances of differentially abundant genera **(A)** and families **(B)** identified by LEfSe analysis. Control: equivalent amount of methanol and ddH_2_O were added into native soil; Atr: native soil spiked with atrazine; Atr-Bio: atrazine-spiked soil with inoculation of strain AT-5; Bio: native soil with inoculation of strain AT-5. Atr-Bio_Bio, comparison of Atr-Bio treatment vs. Bio treatment, Control_Atr, comparison of Control treatment vs. Atr treatment. Early stage, 0–5 days; late stage, 7–14 days.

To identify the biomarkers distinguishing different treatments, LEfSe analysis was used (Supplementary Figure S7). There were 58 potential biomarkers detected at all levels. At the genus level (Figure 5A), the potential biomarkers *Paenarthrobacter* and *Bacillus* were noted in both Bio and Atr-Bio treatments (inoculation treatment). Furthermore, the abundance of *Bacillus* was significantly increased in the inoculation treatment, indicating that inoculation of *Paenarthrobacter* sp. strain AT-5 enriched the genus *Bacillus* in the indigenous microbiome. Meanwhile, the relative abundances of 17 other potential biomarkers including *Marmoricola*, *Nocardioides*, *Agromyces*, and *Solirubrobacter*, decreased in inoculation treatment as compared to that in other treatments. These results indicated that inoculation treatment negatively selected these genera in the indigenous microbiome. It is worth noting that the positively selected biomarkers, including *Marmoricola*, *Nocardioides*, *Agromyces*, and *Solirubrobacter*, were only detected in the atrazine-spiked soil treatment (Atr) at 14 days. They increased markedly in Atr treatment at 14 days as compared to that in 1, 3, and 7 days. These four genera might be the positive biomarkers for atrazine degradation in non-inoculation treatments.

### Bacterial Co-occurrence Networks

**Figure 6:**
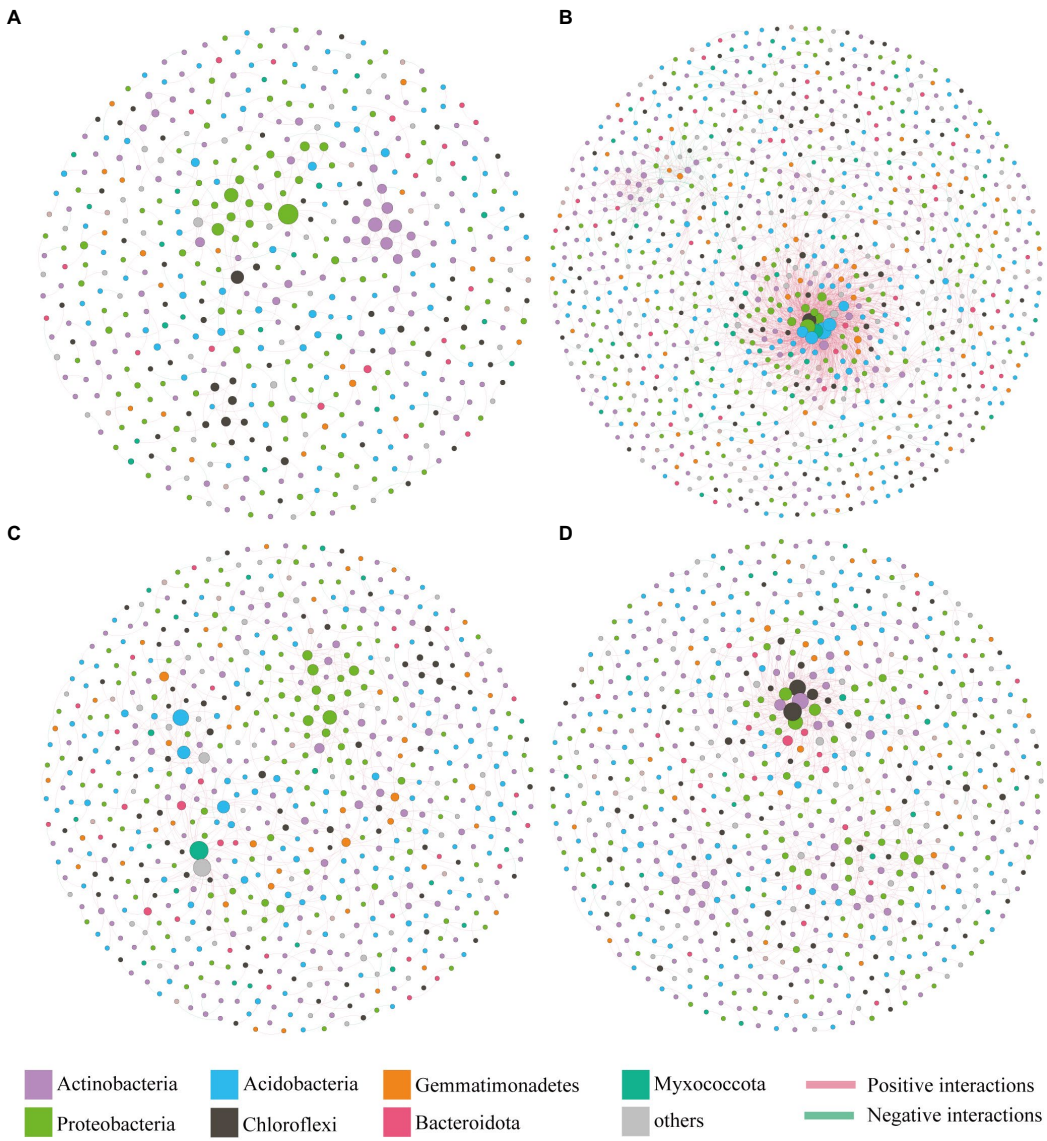
Bacterial co-occurrence networks under different treatments. **(A)** Control; **(B)** Atr; **(C)** Bio; **(D)** Atr-Bio. Control: equivalent amount of methanol and ddH_2_O were added into native soil; Atr: native soil spiked with atrazine; Atr-Bio: atrazine-spiked soil with inoculation of strain AT-5; Bio: native soil with inoculation of strain AT-5.

The co-occurrence patterns of bacterial communities in different treatments (Control, Atr, Bio, and Atr-Bio) were identified through constructing bacterial co-occurrence networks. The topological properties of the co-occurrence patterns varied significantly between the Atr-Bio and Atr networks (Figure 6). The Atr network exhibited more nodes and edges, a higher average degree and average clustering coefficient, as well as higher density and connectedness than other treatment networks, suggesting a much greater complexity and connectedness in the Atr network than that in the Atr-Bio network (Supplementary Figure S8). In addition, positive correlations occupied a dominant position in the all networks, regardless of the different treatments. However, compared to Atr treatments (10.2%), the negative correlations remarkably decreased in the Bio (5.9%) and Atr-Bio (3.5%) treatments, respectively. The higher negative correlations in the Atr network may be attributed to the filter of non-adaptive bacteria by atrazine application. In the Atr-Bio network, the lower negative correlations may be attributed to the removal of atrazine by bioaugmentation, leading to the recovery of the bacterial community.

## Discussion

Microbial communities in natural environments usually do not have the capacity to degrade organic pollutants. Bioaugmentation, a strategy of inoculating specific functional microorganisms for degradation of pollutants, has been proposed as the most potential method to clean up pollutant-contaminated sites (Huang et al., 2019; Yang et al., 2021). Though some previous studies have investigated the bioaugmentation of atrazine-contaminated sites with atrazine-degrading strains, there are few studies about the effect of bioaugmentation on indigenous soil microbial communities (Wang et al., 2013). Nevertheless, the inoculation of exogenous strains may significantly change the structure of soil microbiome and affect its potential function (Yang et al., 2021). To determine whether bioaugmentation has negative environmental effects or not, the interactions between the inoculated exogenous strains, pollutants, and soil microbial consortia were required to be investigated. In this study, *Paenarthrobacter* sp. AT-5 was inoculated for atrazine removal to investigate the effects of bioaugmentation on the soil microbiome and the reconstructing process of microbial community.

In this study, the removal rate of atrazine by bioaugmentation of strain AT-5 was 95.9% at 7 days, Gao et al. (2018) found the half-life of atrazine in soil treated with *Arthrobacter* sp. strain HB-5 was significantly reduced to 6.3 days (Gao et al., 2018), showing that strain AT-5 has an excellent potential for atrazine degradation in soils. In addition, successful bioaugmentation relies not only on the degradability of the inoculum but also on its ability to survive in the environment (Singer et al., 2005; Chi et al., 2013). Some studies have demonstrated that the persistence of inoculum in the environment is a key factor of bioaugmentation (Chi et al., 2013; Yang et al., 2021). Our previous study showed the inoculated exogenous degrading strain could not survive well in soil, resulting in the decrease of chlorpyrifos mineralization rate (Jia et al., 2021a). By qRT-PCR and 16S rRNA-amplicon sequencing, we found that the inoculated strain AT-5 survived well in the soil and remained relatively stable during the incubation period of 14 days, which ensured its remediation efficiency for atrazine-contaminated soils. In addition, bioremediation is often subject to environmental constraints, such as soil type (Jia et al., 2021a). However, strain AT-5 showed good remediation effects in three different soils collected from Jining, Langfang, and Xuzhou, China (Supplementary Figures S3, S4), indicating that strain AT-5 has great potentials for the remediation of atrazine-contaminated different types of soil.

Actinobacteriota and Proteobacteria were the most abundant bacterial phyla in all treatments in our study. Previous studies found that Proteobacteria are the dominant microorganisms in various pesticide-contaminated soils due to their good tolerance to pollutants (Vaishampayan et al., 2007; Jia et al., 2021b). In addition, several atrazine-degrading bacteria have been identified in Actinobacteriota and Proteobacteria, such as *Arthrobacter/Paenarthrobacter* (Vaishampayan et al., 2007), *Pseudomonas* (de Souza et al., 1998), and *Nocardioides* (Piutti et al., 2003). Compared with the control treatment, the abundance of *Bacillus* significantly increased in the inoculation treatment, indicating that the potential indigenous *Bacillus* may be directly or indirectly involved in the degradation of atrazine. Up to now, several *Bacillus*, such as *Bacillus licheniformis* ATLJ-5 (Zhu et al., 2019), *Bacillus megaterium* ATLJ-11 (Zhu et al., 2019) and *Bacillus subtilis* HB-6 (Wang et al., 2014) have been reported to be capable of degrading atrazine and its metabolites. Hence, the addition of atrazine increased the relative abundance of potential biomarkers such as *Bacillus*, which may be involved in the biodegradation of atrazine or its metabolites.

Bacterial richness and diversity significantly decreased in the inoculation treatments, which were also observed during the bioaugmentation of acetamiprid-contaminated soil with *Pigmentiphaga* sp. strain D-2 (Yang et al., 2021). This phenomenon observed in our study could be attributed to the persistence and niche occupation of strain of AT-5. Moreover, inoculation of strain AT-5 enhanced atrazine degradation, intermediate production, and nutrient consumption in soil. These changes in the microenvironment may also lead to a significant reduction in bacterial richness and diversity (Yang et al., 2021). However, there was no significant difference in richness and diversity index between the control treatment (methanol and ddH_2_O application) and Atr treatment (atrazine application). These results indicated that the main driving factor for the change of bacterial community structure in the inoculation treatments is the addition of strain AT-5. It has been reported that the abundance of inoculum decreased after elimination of pollutants in soils (Cunliffe and Kertesz, 2006; Niu et al., 2009; Chi et al., 2013). Considering the influence of complex environmental factors in soils and the competition between inoculum and indigenous microorganisms, we speculate that strain AT-5 might not be able to maintain high abundance in *in-situ* soils after elimination of atrazine. Unfortunately, we did not collect soil samples from the Atr-Bio treatment on a longer time scale. Therefore, it is needed to clarify the final fate of strain AT-5 in soils in future studies.

## Data Availability Statement

The original contributions presented in the study are included in the article/Supplementary Material, further inquiries can be directed to the corresponding authors.

## Author Contributions

KC and XX conceived and designed the experiments. WJ and TY performed the experiments. XX and WJ analyzed the data. WJ, NL, and WD prepared the manuscript. JJ, KC, and XX revised the manuscript. All authors contributed to the article and approved the submitted version.

## Funding

This work was financially supported by the grant of National Key R&D Program of China (2018YFA0901200), the National Natural Science Foundation of China (31870095 and 41977120), and the China Agriculture Research System of MOF and MARA.

## Conflict of Interest

The authors declare that the research was conducted in the absence of any commercial or financial relationships that could be construed as a potential conflict of interest.

## Publisher’s Note

All claims expressed in this article are solely those of the authors and do not necessarily represent those of their affiliated organizations, or those of the publisher, the editors and the reviewers. Any product that may be evaluated in this article, or claim that may be made by its manufacturer, is not guaranteed or endorsed by the publisher.
