# Supplementary material for: Bioaugmentation of Atrazine-Contaminated Soil With Paenarthrobacter sp. Strain AT-5 and Its Effect on the Soil Microbiome
Source: Front Microbiol. 2021 Dec 8;12:771463. doi: 10.3389/fmicb.2021.771463 (PMC8692732; doi:10.3389/fmicb.2021.771463)
Supplement: Supplementary file 1 [file Table_1.DOCX]

Table S1. Properties of soil used in this study.

| **Soil properties** | **Results** |
| --- | --- |
| Organic carbon content (g kg^-1^) | 3.23 |
| pH (H_2_O) | 7.23 |
| Water-holding capacity (%) | 56 |
| Total phosphorus (g kg^-1^) | 0.47 |
| Total potassium (g kg^-1^) | 11.03 |
| Total nitrogen (g kg^-1^) | 0.39 |


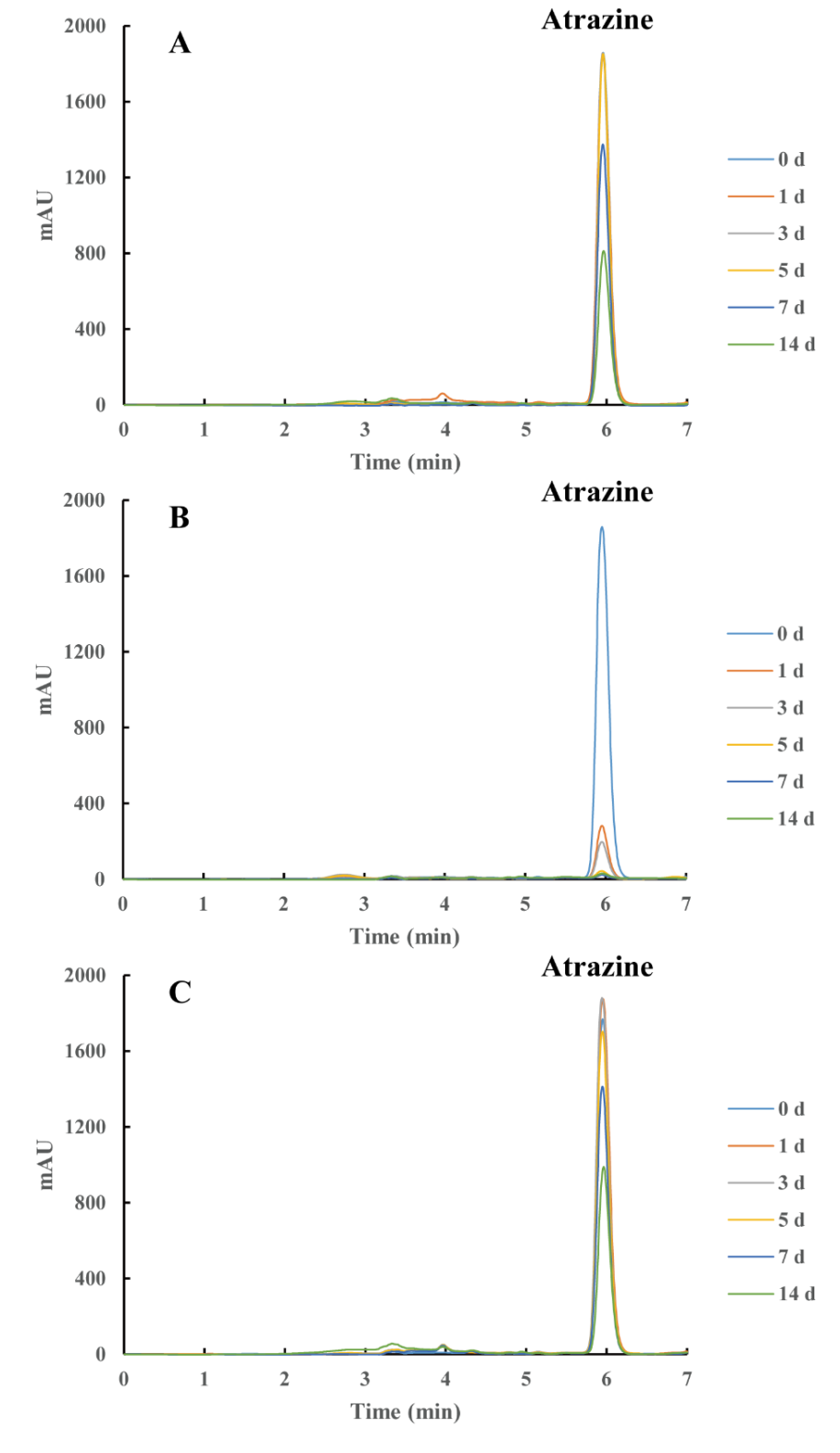


Figure S1. HPLC analysis of the dynamic changes of atrazine residues in soils. A, Control: native soil spiked with atrazine; B, Bioaugmentation: atrazine-spiked soil with inoculation of strain AT-5; C, Sterilized soil: sterilized soil spiked with atrazine.


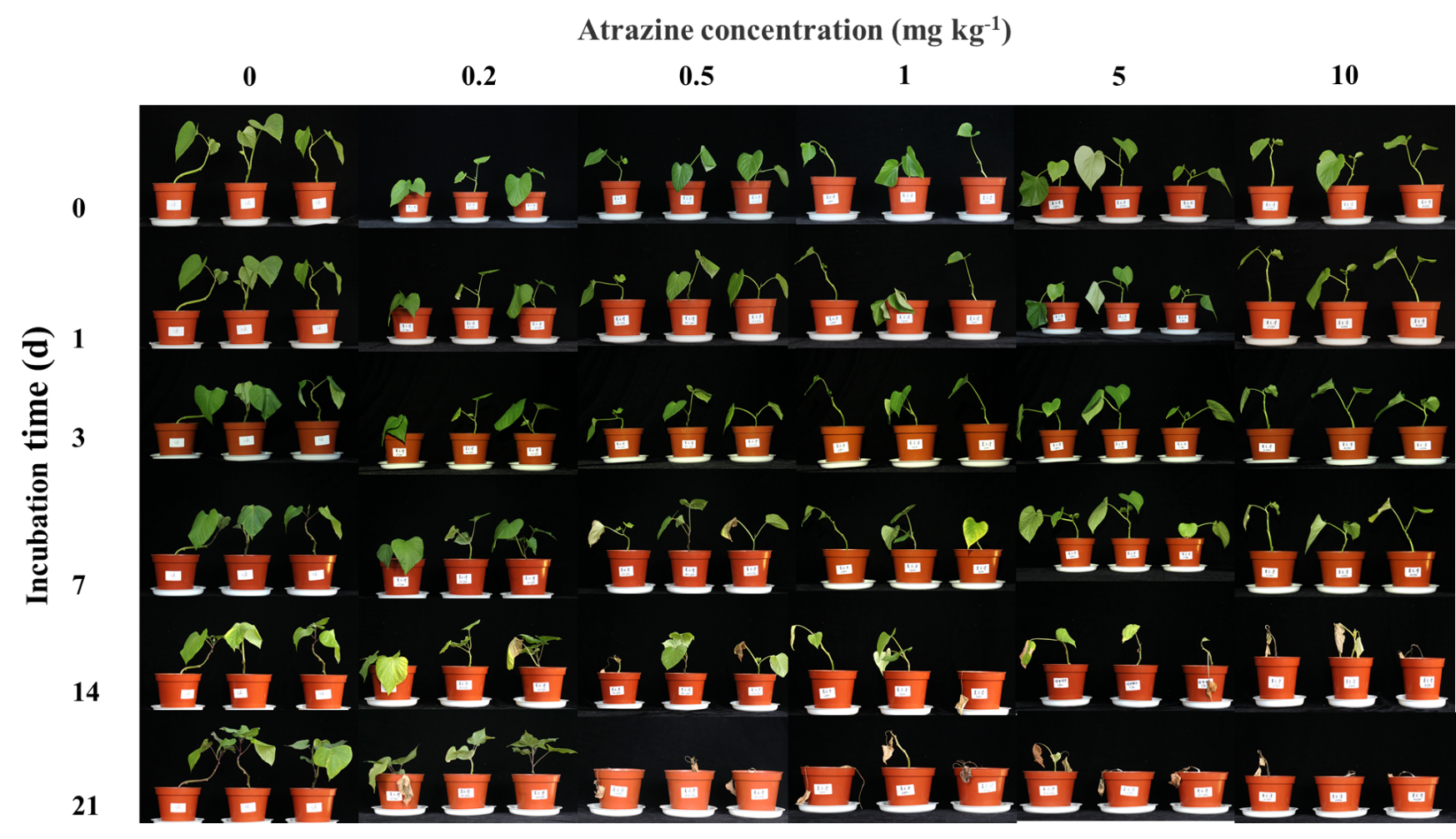


Figure S2. The phytotoxicity of atrazine residues in soil on sweet potato seedlings.


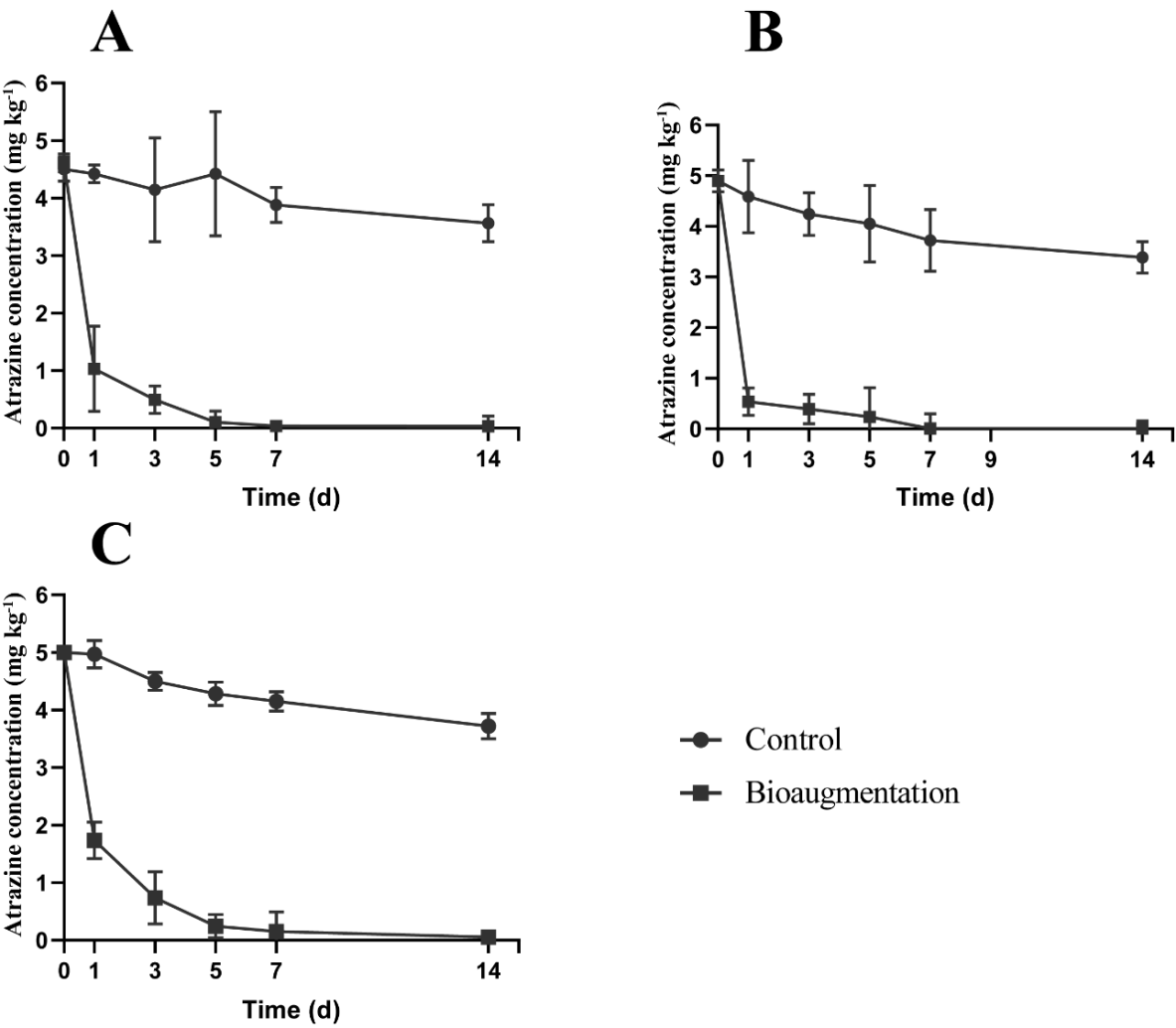


Figure S3. Dissipation of atrazine in three different soils. (A), (B) and (C) represent soils from Jining, Langfang and Xuzhou, China.


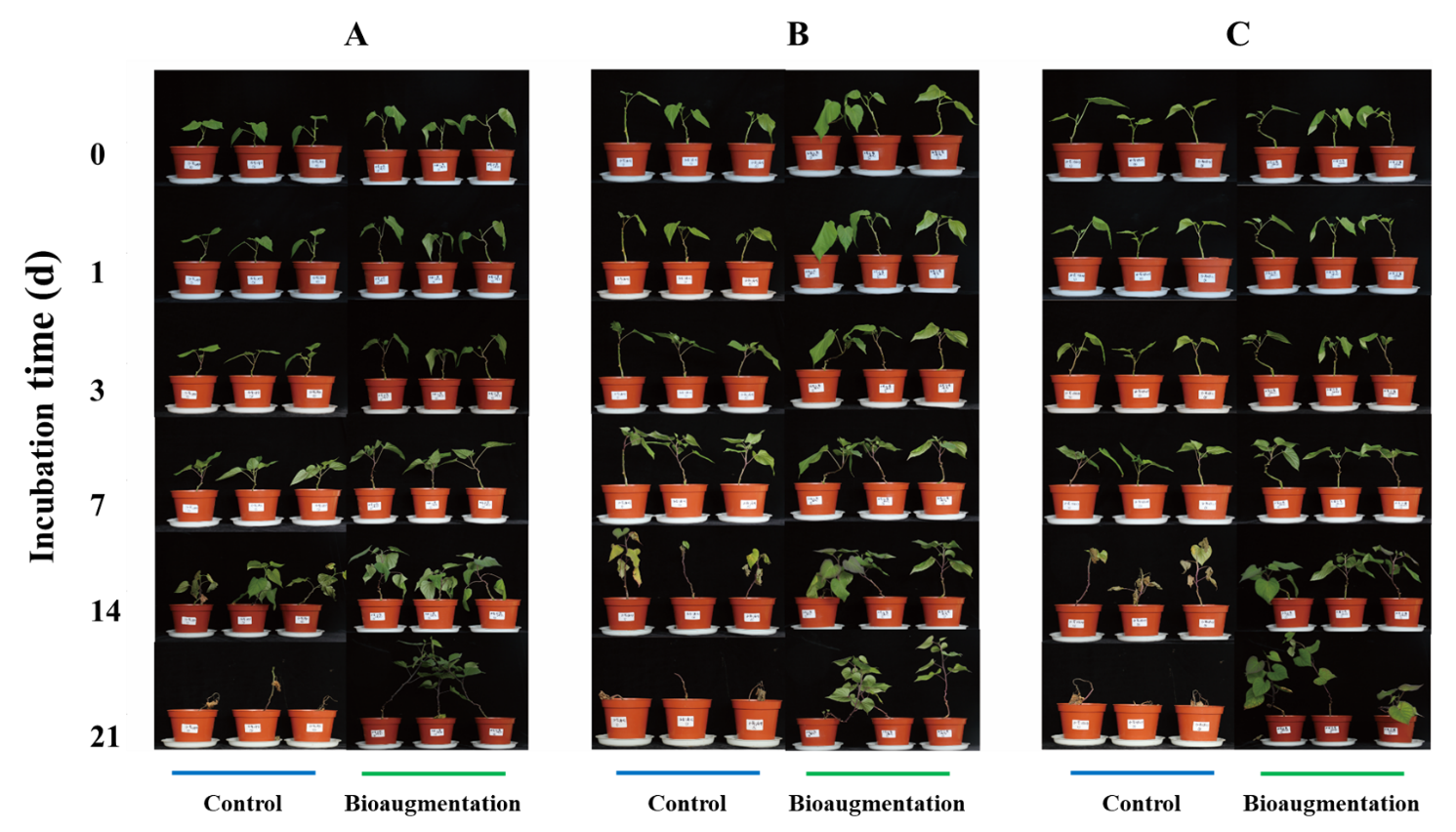


Figure S4. The phytotoxicity of atrazine on sweet potato seedlings alleviated by bioaugmentation in soils collected from Jining (A), Langfang (B) and Xuzhou (C). Control: native soil spiked with atrazine after incubated 14 days, and the concentrations of atrazine remaining in soils were 3.56±0.31 mg kg^-1^ (A), 3.38±0.32 mg kg^-1^ (B) and 3.71±0.21 mg kg^-1^ (C), respectively. Bioaugmentation: atrazine-spiked soil with inoculation of strain AT-5 after incubated 14 days, and the concentrations of atrazine remaining in soils were 0.03±0.0.007 mg kg^-1^ (A), 0.08±0.009 mg kg^-1^ (B) and 0.05±0.01 mg kg^-1^ (C), respectively. Three replicates were set for each treatment.


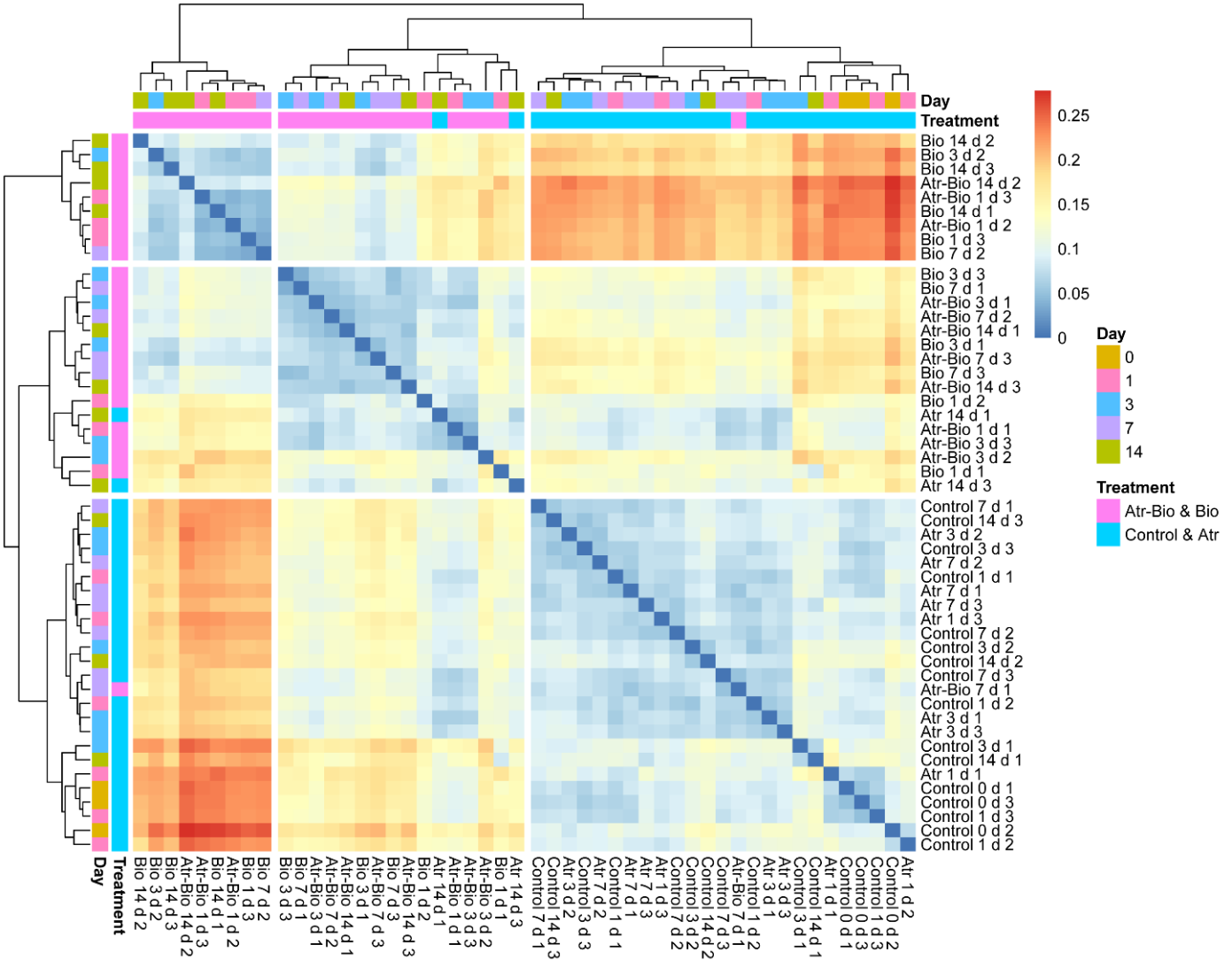


Figure S5. Heatmap with weighted UniFrac distances comparing bacterial communities among the treatments. Control: equivalent amount of methanol and ddH_2_O were added into native soil; Atr: native soil spiked with atrazine; Atr-Bio: atrazine-spiked soil with inoculation of strain AT-5; Bio: native soil with inoculation of strain AT-5.


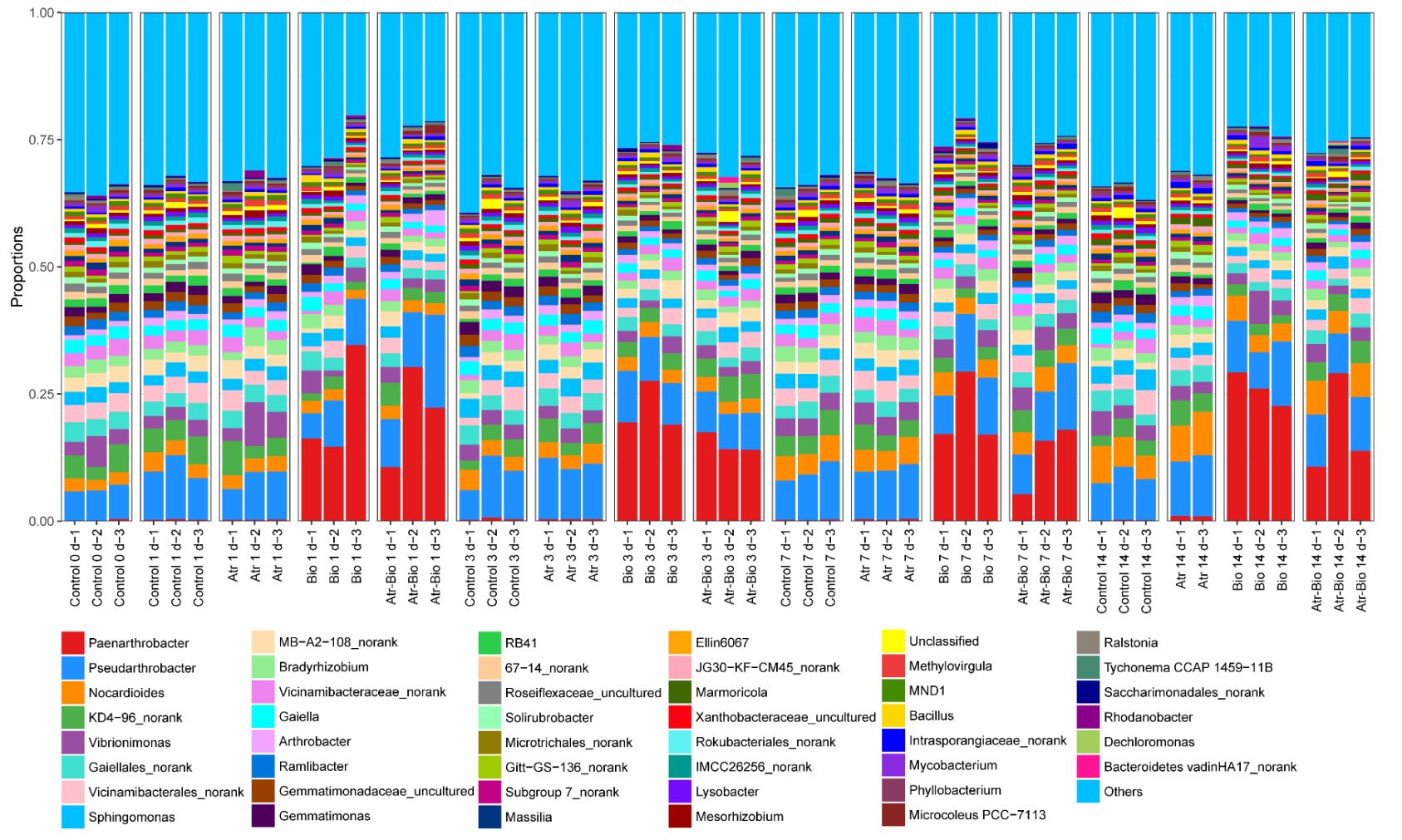


Figure S6. The microbial community composition at the genus level under different treatments. Control: equivalent amount of methanol and ddH_2_O were added into native soil; Atr: native soil spiked with atrazine; Atr-Bio: atrazine-spiked soil with inoculation of strain AT-5; Bio: native soil with inoculation of strain AT-5.


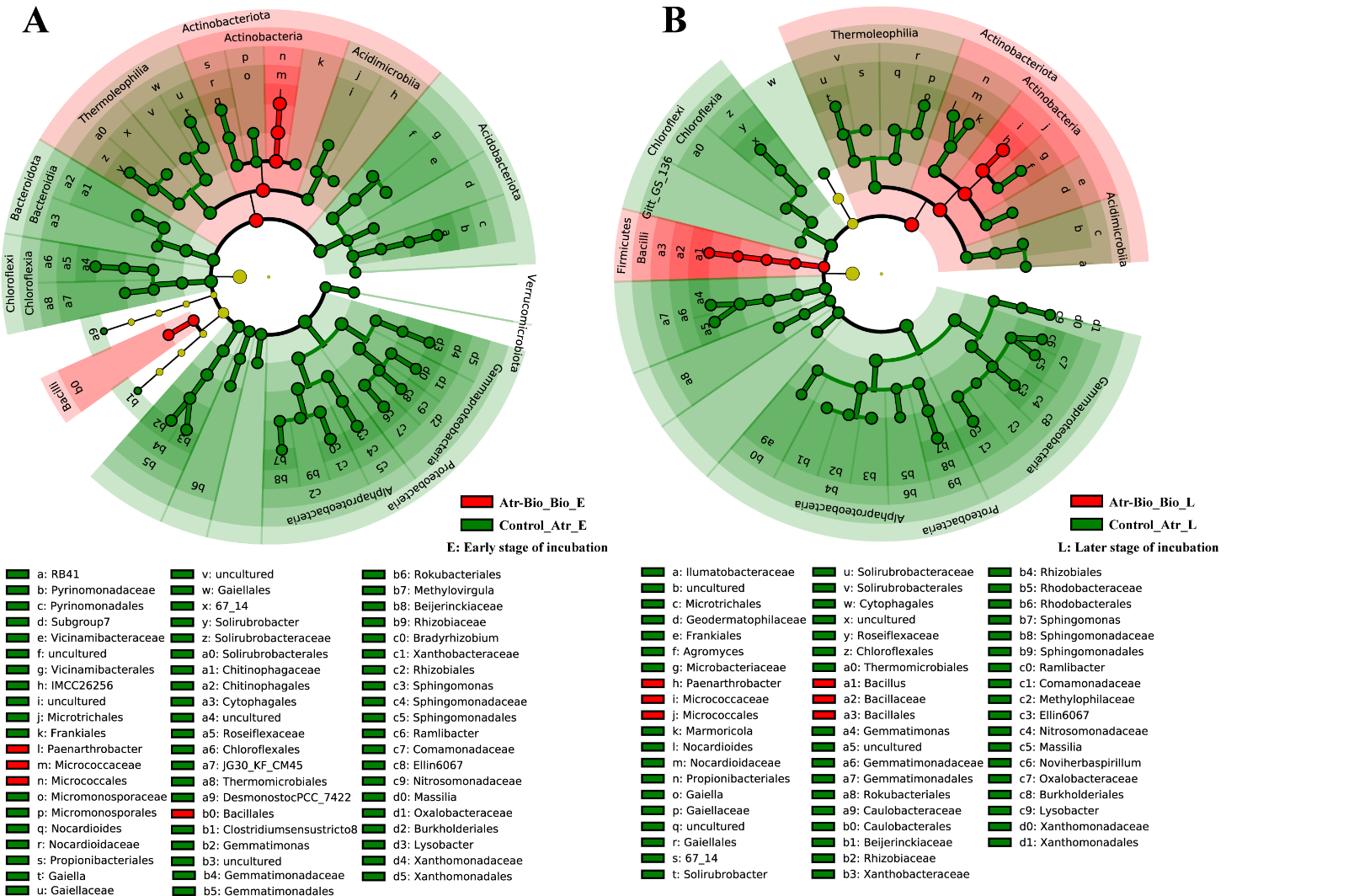


Figure S7. LEfSe cladogram illustrating taxonomic differences in the bacterial communities and indicating the most differentially abundant taxa among treatments (A, Early stage, 0-5 days; B, late stage, 7-14 days).


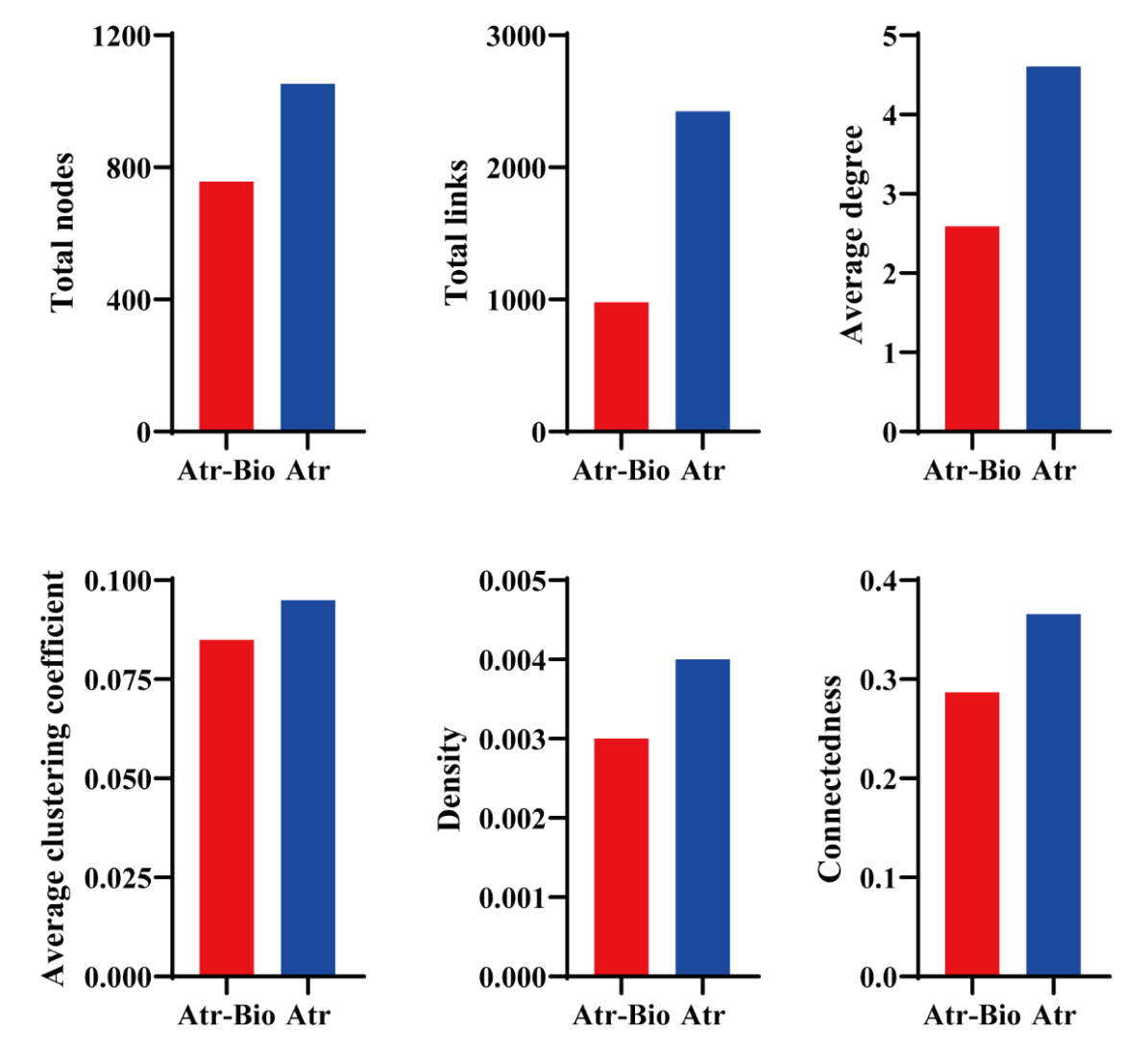


Figure S8. Variations in the global network properties. Atr-Bio: atrazine-spiked soil with inoculation of strain AT-5; Atr: native soil spiked with atrazine.
